# The complete mitochondrial genome of the jumping plant bug *Halticus minutus* Reuter, 1885 (Hemiptera: Miridae)

**DOI:** 10.1080/23802359.2022.2113748

**Published:** 2022-08-30

**Authors:** Chunyan Zhong, Meihua Huang, Keqing Li, Hongda Zou, Lifei Huang

**Affiliations:** a Zhaoqing Institute of Agricultural Sciences, Zhaoqing, China; b Crop Research Institute, Guangdong Academy of Agricultural Sciences, Guangdong Provincial Key Laboratory of Crops Genetics and Improvement, Guangzhou, China

**Keywords:** *Halticus minutus*, complete mitochondrial genome, phylogenetic analysis

## Abstract

The complete mitochondrial genome of *Halticus minutus* was sequenced and analyzed in this study. The mitochondrial genome is 15,403 bp in size and comprises 13 protein-coding genes, 22 tRNA genes, two rRNA genes, and one control region (D-loop). The nucleotide composition of the mitogenome is 41.81% A, 32.50% T, 10.43% G, and 15.26% C. Despite only a few references available on the complete mitochondrial genome of Miridae, phylogenetic analysis suggested that *H. minutus* is most closely related to *Nesidiocoris tenuis*.

Sweet potato (*Ipomoea batatas* (L.) Lam.) is a major dry grain and feed crop worldwide, which also serves as industrial and food raw material and horticultural crop. China is the largest producer of sweet potatoes globally. The annual planting area of sweet potato was 2.4 million hectares (Wang et al. 2021). The jumping plant bug *Halticus minutus* Reuter, 1885 (Hemiptera: Miridae) is widely distributed in Asia (Amalin and Vasquez 1993; Eyles 2005). *Halticus minutus* has diverse host plants, including sweet potato, bean, corn, wheat, barley, eggplant, tobacco, cotton, and alfalfa (Henry 1983; Wu and Yang 1987). In China, *H. minutus* is one of the most common sweet potato pests and is considered an economically important pest in sweet potato and soybean (Tong and Wang 1987). However, genomic information of *H. minutus* is scanty. In the present study, we sequenced the intact mitochondrial genome (mitogenome) of *H. minutus*. Further, we characterized the complete mitochondrial genome and determined its phylogenetic relationship with other close species. The findings of this study provide a basis for future molecular studies of *H. minutus.*

Adults of *H. minutus* were collected using a sweep net from sweet potato fields in Zhaoqing Institute of Agricultural Sciences, Zhaoqing, Guangdong Province (23°10′40.6524″N, 112°34′32.7288″E), China. Voucher specimens were deposited in the Crop Research Institute, Guangdong Academy of Agricultural Sciences (contact Lifei Huang, email: hlf157@163.com) under the voucher number 20201022. The whole genome of 10 *H. minutus* was extracted by DNeasy Blood & Tissue Kit (QIAGEN, Hilden, Germany). Then, paired-end libraries of 150 bp were constructed and sequenced on the Novaseq System (Illumina, San Diego, CA). The mitochondrial genome was assembled using SPAdes v3.10.1 as described previously (Bankevich et al. 2012). Additionally, SSPACE v2.0 (Boetzer et al. 2011) was applied to connect the contig sequence to obtain scaffolds. Gapfiller v2.1.1 (Boetzer and Pirovano 2012) was also utilized to fill gaps. The mitogenome sequences were annotated on the Mitos2 website (http://mitos2.bioinf.uni-leipzig.de), and then adjusted manually.

The complete mitochondrial genome of *H. minutus* consists of double-stranded circular molecules with a length of 15,403 bp. It also contains 13 protein-coding genes, 22 tRNA genes, and two rRNA genes. The nucleotide composition of the complete mtDNA is 74.31% A + T and 25.69% G + C, indicating a significant A and T bias. Phylogenetic analysis was performed using 18 complete mitochondrial genomes, including *Corythucha ciliata* (Say, 1832) (Hemiptera: Tingidae) and *C. marmorata* (Uhler, 1878) (Hemiptera: Tingidae) as outgroups. The complete mitochondrial genomic sequences among the species were aligned using MAFFT. The best partition scheme and substitution models were identified using PartitionFinder v1.1.1 (Lanfear et al. 2012). The maximum-likelihood (ML) tree was constructed using RAXML8.2.9 (Stamatakis 2014) with 1000 bootstraps. GRT was selected as the best-fit model according to Modeltest3.7 (Posada and Crandall 1998). The Bayesian analysis was also performed using MrBayes 3.2 (Ronquist et al. 2012). Based on the phylogenetic tree analysis, the mitochondrial sequences of *H. minutus* formed a single clade (Figure 1), indicating a relatively close relationship with the clade formed by two mitochondrial sequences of *Nesidiocoris tenuis* (Reuter, 1895) (Heteroptera: Miridae).

**Figure 1.**
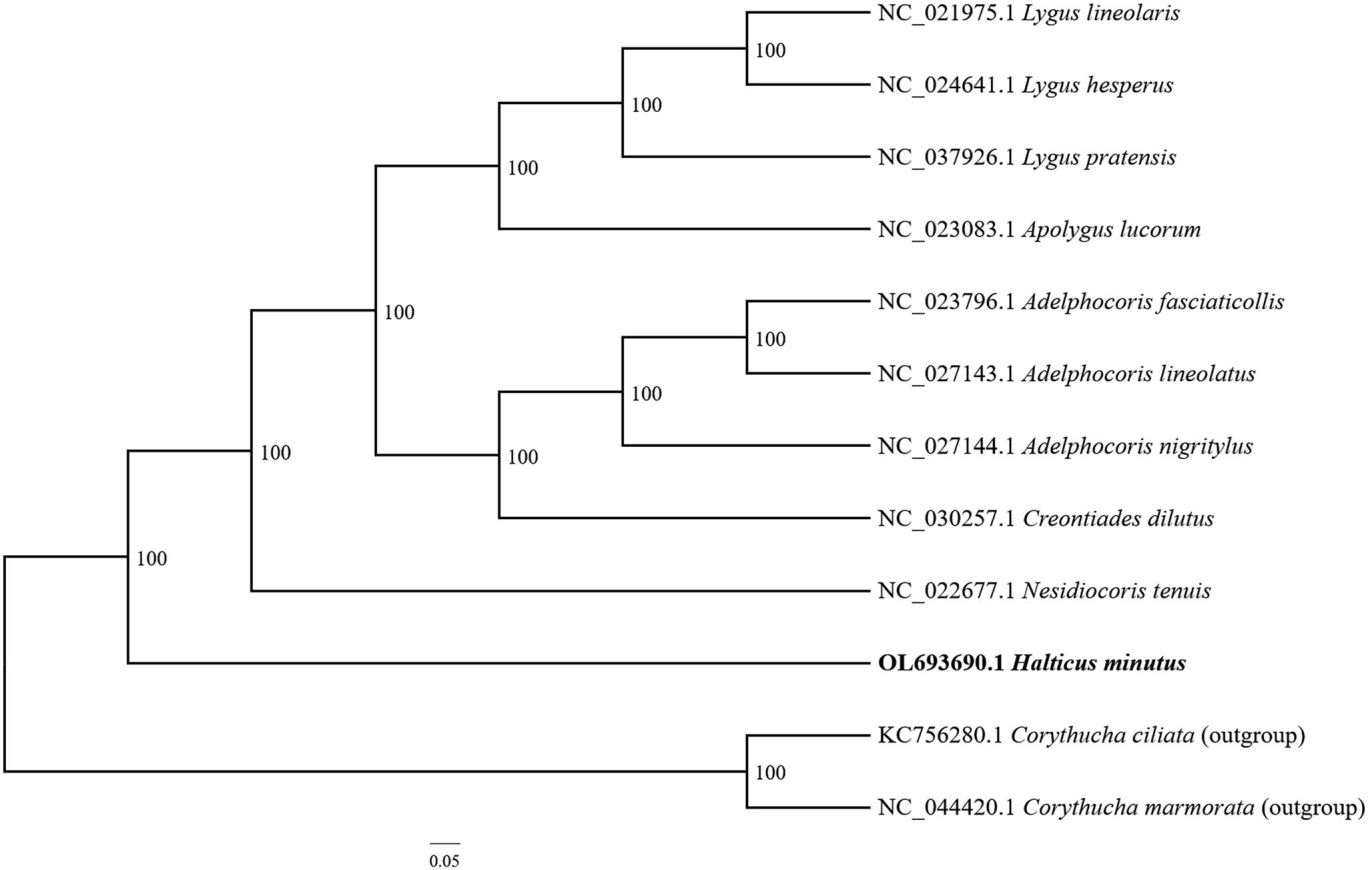
The phylogenetic tree based on 18 complete mitochondrial genome sequences. Numbers indicate the Bayesian posterior probabilities.

## Data Availability

The data that support the findings of this study are openly available in GenBank of NCBI at https://www.ncbi.nlm.nih.gov, reference number OL693690. The associated BioProject, BioSample, and SRA numbers are PRJNA809507, SAMN26182809, and SRR18131583, respectively.
